# The effects of maternal alcohol consumption during pregnancy on adverse fetal outcomes among pregnant women attending antenatal care at public health facilities in Gondar town, Northwest Ethiopia: a prospective cohort study

**DOI:** 10.1186/s13011-021-00401-x

**Published:** 2021-08-26

**Authors:** Alemu Earsido Addila, Telake Azale, Yigzaw Kebede Gete, Mezgebu Yitayal

**Affiliations:** 1 Department of Public Health, College of Medicine and Health Sciences, Wachemo University, Hossana, Ethiopia; 2 Department of Epidemiology and Biostatistics, College of Medicine and Health Sciences, Institute of Public Health, University of Gondar, Gondar, Ethiopia; 3 Department of Health Education and Behavioral Sciences, College of Medicine and Health Sciences, Institute of Public Health, University of Gondar, Gondar, Ethiopia; 4 Department of Health Systems and Policy, College of Medicine and Health Sciences, Institute of Public Health, University of Gondar, Gondar, Ethiopia

**Keywords:** Pregnant women, Alcohol use, Adverse health outcomes, Ethiopia

## Abstract

**Background:**

The teratogenic effect of fetal alcohol exposure may lead to actual and potential problems, instantly after birth, at infancy; or even later, and mental impairment in life. This study aimed to investigate the effects of maternal alcohol consumption during pregnancy on adverse fetal outcomes at Gondar town public health facilities, Northwest Ethiopia.

**Methods:**

A facility-based prospective cohort study was performed among 1778 pregnant women who were booked for antenatal care in selected public health facilities from 29 October 2019 to 7 May 2020 in Gondar town. We used a two-stage random sampling technique to recruit and include participants in the cohort. Data were collected using the Alcohol Use Disorders Identification Test – Consumption (AUDIT-C) standardized and pre-tested questionnaire. Multivariable analysis was performed to examine the association between reported prenatal alcohol exposure (non-hazardous and hazardous) and interested adverse birth outcomes using log-binomial regression modeling. The burden of outcomes was reported using the adjusted risk ratio and population-attributable risk (PAR).

**Results:**

A total of 1686 pregnant women were included in the analysis, which revealed that the incidences of low birth weight, preterm, and stillbirth were 12.63% (95% CI: 11.12, 14.31), 6.05% (95% CI: 5.00, 7.29) and 4.27% (95% CI: 3.4, 5.35), respectively. Non-hazardous and hazardous alcohol consumption during pregnancy was significantly associated with low birth weight (ARR = 1.50; 95% CI: 1.31, 1.98) and (ARR = 2.34; 95% CI: 1.66, 3.30), respectively. Hazardous alcohol consumption during pregnancy was also significantly associated with preterm birth (ARR = 2.06; 95% CI: 1.21, 3.52). The adjusted PAR of low birth weight related to non-hazardous and hazardous alcohol drinking during pregnancy was 11.72 and 8.44%, respectively. The adjusted PAR of hazardous alcohol consumption was 6.80% for preterm.

**Conclusions:**

Our findings suggest that there is an increasing risk of adverse birth outcomes, particularly preterm delivery and low birth weight, with increasing levels of alcohol intake. This result showed that the prevention of maternal alcohol use during pregnancy has the potential to reduce low birth weight and preterm birth. Hence, screening women for alcohol use during antenatal care visits and providing advice with rigorous follow-up of women who used alcohol may save the fetus from the potential risks of adverse birth outcomes.

## Background

Alcohol consumption during pregnancy may have adverse effects not only on the incidence of diseases, injuries, and other health conditions to the women but also on the infants and children [1]. Pregnant women may consume alcohol without fully understanding the ill effects of alcohol consuming [2]. Since alcohol passes through the placental, fetal blood may have the same blood alcohol concentration or higher than that of the mother that can result in various adverse effects on the fetus besides the risk of harm to the mother [3]. The body of the fetus during the developmental stage does not similarly process alcohol an adult does; the alcohol is more concentrated in the body of the fetus, and it can prevent the passage of adequate amount of nutrition and oxygen to the vital organs of the fetus [4]. Subsequently, the teratogenic effects of fetal alcohol exposure may lead to actual and potential problems, instantly after birth, at infancy, or even later, leading to anatomical abnormalities, behavioral problems, and mental impairment in life [5]. On the other hand, a wide range of birth defects termed fetal alcohol spectrum disorder (FASD) has been associated with alcohol use during pregnancy [6–8].

The degree of effects of alcohol use during pregnancy may vary depending on the frequency of exposure to alcohol, dose, duration, genetic factors, maternal nutrition, and developmental stage of the fetus at exposure [3, 9–12]. Due to genetic and lifestyle factors, there may also have different outcomes from the same exposure [13, 14]. However, there is no currently assured exact dose-response relationship between the amount of alcohol consumed during the pregnancy and the degree of the problem or a risk threshold caused by alcohol in the infant [15]. According to different studies, nobody knows the exact amount of alcohol that is potentially harmful to the developing baby in any trimester. Hence, researchers and health professionals recommend not drinking any amount of alcohol for pregnant women as well as women who are trying to get pregnant [16–23]. The consequences and safety of low-to-moderate alcohol consumption during pregnancy on the fetus is still inconclusive and discordant [9, 15, 24, 25].It is argued that the lack of agreement between studies might be due to heterogeneity of the study participants, methodological differences, low statistical power, potential confounding factors, and the difference in detecting tools used or biased information on maternal alcohol consumption [23, 26]. On the other hand, multiple adverse birth outcomes have been correlated with hazardous alcohol use during pregnancy, including low birth weight, preterm birth, intrauterine growth retardation (IUGR), having low weight for head circumferences, and small for gestational age (SGA) [9, 25, 27].

Despite many guidelines that advise that women should avoid drinking any alcoholic beverages during any stage of pregnancy to save future generations from alcohol-associated mental, physical, and behavioral abnormalities, numerous studies have shown that a significant number of pregnant women continue to drink alcohol in Ethiopia [28–31]. Regardless of a high proportion of pregnant women consume alcoholic beverages; policies have paid little attention to risks associated with alcohol consumption during pregnancy.

In Ethiopia, due to the rapid expansion of industrially-manufactured newly branded alcoholic beverages over time and the rising purchasing power of the society [32], a great proportion of pregnant mothers consume alcoholic beverages [28–31]. Moreover, homemade indigenous alcoholic beverages such as *Tella* (traditional Ethiopian beer fermented from mostly barley but also with wheat, maize, sorghum, and mixed with ‘*Gesho’* [Rhamnusprinioides]) [33], *Areki* (a whiskey-like drink distilled from fermented barley or maize and mixed with [Rhamnusprinioides]), and *Tej* (a honey wine), *Borde*, and *Korofe* are generally common in Ethiopia and everyone drinks without any confinement of official body [34].

Previously conducted studies in different parts of the world had an inconsistent association between prenatal alcohol exposure and adverse fetal outcomes to take appropriate interventions [9, 35–39]. Therefore, this study focused on determining the effects of alcohol use during pregnancy on adverse fetal outcomes such as preterm, stillbirth, and low birth weight, as they are one of the major causes of neonatal morbidity and mortality in low- and middle-income countries [40], including Ethiopia [41–43]. By investigating the effects of alcohol consumption during pregnancy, the present study could make a novel share to help fill the gaps in the current literature and update future guidelines concerning alcohol consumption during pregnancy.

## Materials and methods

### Study design, period, and study setting

We carried out a facility-based prospective cohort study among pregnant women who were booked for antenatal care in selected public health facilities from 29 October 2019 to 7 May 2020 in Gondar town. The included health facilities were one hospital (University of Gondar Comprehensive Specialized Hospital) and three health centers (Gondar polyclinic, Azezo, and Maraki). Gondar town is located about 727 km far from Addis Ababa, the capital city of Ethiopia. According to the Gondar town Finance and Economic Development branch Office report in 2018, the total population of Gondar town was approximately 338, 646 (165, 937 males and 172, 709 females). Of these females, 7454 were estimated to be pregnant. In the town, there are eight health centers and one comprehensive specialized hospital [44].

### Sample size determination and sampling procedure

**Fig. 1:**
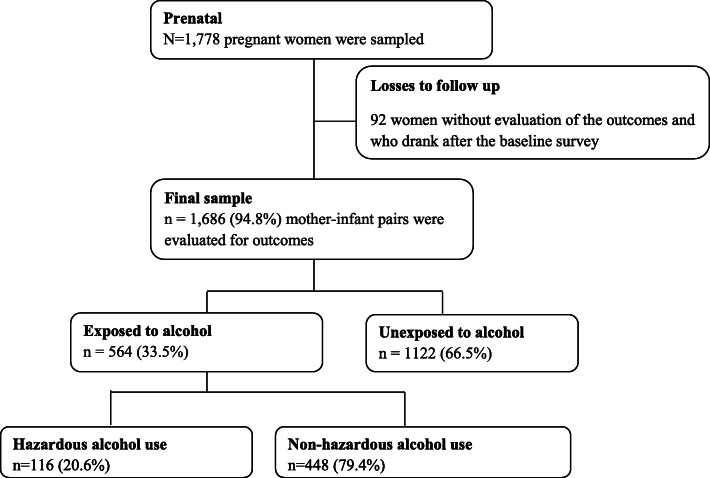
Flowchart of the study participants at Gondar town public health facilities, Northwest Ethiopia, 2020

The sample size was determined by using EPI INFO version 7.2.1.0 STAT CALC software cohort study as described by Fleiss with continuity correction to estimate the sample size (https://silo.tips/download/statcalc-calculating-a-sample-size-with-epi-info) [45]. We used the following assumptions: two-sided 95% confidence level, power of 80%, the ratio of sample size 2:1 to detect the odds ratio of 1.9 by considering 6.4% of low birth weight in the unexposed group and 11.7% in the exposed group to bring a difference in two population based on the research conducted in Brazil [46]. These rates were taken from a study conducted in another country because we did not find similar studies in Ethiopia or other similar situations. Finally, 1778 study participants (593 exposed and 1185 unexposed to alcohol use) were enrolled using a design effect of 1.5 and 10% withdrawn or attrition rate from the cohort for a variety of reasons. We used a two-stage random sampling technique to recruit pregnant women and include them in the cohort. In the first stage, we applied simple random sampling to select three health centers. In addition to these three health centers, one hospital was purposively included in the study. In the second stage, pregnant mothers who fulfilled the inclusion criteria were chosen using a systematic sampling technique. The sample size was proportionally allocated to each health facility based on previous client-flow information. A flow diagram of the study participants was presented in **(**Fig. 1**)**.

### Study variables

#### The outcome of interest

Information on birth outcomes was obtained from health facilities’ maternity records and interviewing the mothers. The outcome interest variables of the study were preterm, stillbirth, and low birth weight which were categorized as a dichotomous variable (yes/no); we used the World Health Organization (WHO) recommended definition for international comparisons for the outcomes. Birth weight was obtained from the delivery logbook to categorize infants as low birth weight (< 2500 g) regardless of gestational age. The estimated time of conception and subsequent gestational age at delivery was calculated based on the first day of the last menstrual period (LMP) or an ultrasound estimated result. Preterm delivery and stillbirth were defined as babies born before 37 weeks of pregnancy and a baby born with no signs of life at or after 28 weeks of gestation, respectively. Alcohol consumption during pregnancy was the main independent variable.

#### Potential confounding variables

Data were collected at baseline and postnatal on various risk factors through the pregnancy, including comprehensive maternal characteristics and potential confounding variables. Besides, detailed pregnancy history was gathered, including pre-existing medical conditions. Some of the included potential confounding variables were socio-demographic characteristics: maternal age, religion, ethnicity, household wealth status, education of respondents, education of husband, marital status and occupation; obstetrics and some medical factors: parity, history of abortion (bleeding during pregnancy), history of preterm birth, history of stillbirth, unwanted pregnancy, hypertensive disorders in pregnancy [47](chronic hypertension, preeclampsia-eclampsia, preeclampsia superimposed on chronic hypertension, and gestational hypertension), gestational diabetes, Mid-Upper Arm Circumference (MUAC), and sex of infant and behavioral variables: cigarette smoking, coffee intake, khat chewing were evaluated.

### Participant selection and recruitment

Women were enrolled and a baseline interview was executed if they were in the first 2 weeks of the third trimester or 28th weeks of gestational age. Because if the baseline data were collected and study participants were advised to stop alcohol intake in the first or/and second trimesters, they might not give the right information about alcohol use in the next interview that may underestimate and lead to false-negative outcomes. The postpartum interview was carried out following delivery, typically in the health facility during the postpartum stay or within 48 h of delivery. All mother-newborns pairs in Gondar town were a source population, and pregnant women who were sampled in the selected health facilities were the study participants. Medical health facility documents or log booklets for infants and mothers were reviewed to collect necessary information related to delivery, selected medical risk factors, and some potential confounders. The eligible study participants in the follow-up were restricted to a singleton pregnancy and aged 18 years or above. If the birth outcomes were not well known due to giving birth at their home or incomplete registration, they were excluded from the study. If a woman was uncertain in remembering the first day of the last menstrual period, the cycle was irregular or there was a difference of more than 7 days, and a booking ultrasound scan estimate was not preferred, they were also excluded from the study.

### Exposure ascertainment and alcohol use measures

To ascertain fetal alcohol exposure, we used maternal self-reporting that is the most common clinical instrument and standard in detecting alcohol exposure [48]. Information on alcohol intake during pregnancy was collected for specific trimesters of pregnancy during baseline prenatal and postpartum interviews. During the baseline interview, study participants were asked in detail about alcohol use information during the first and second trimesters, including the type and quantity of alcoholic beverages. Drinking information of the third trimester was collected in the postpartum interview. For each type of alcoholic beverage, pregnant women were asked how often they consumed alcohol and the number of drinks they drank during the specific trimester based on the AUDIT-C questionnaire [49–51].AUDIT-C is the most popular shortened version of the 10-item AUDIT that comprises three items to assess alcohol consumption cross-culturally and identify hazardous drinkers [49, 52]. The tool had shown to be a valid instrument for alcohol consumption since pregnancy recognition based on self-report [53]. The questionnaire was adjusted by considering the local context of alcoholic beverages of alcohol content and drinking containers. The amount of alcohol content in a standard drink varies from country to country; we used the WHO’s standard for this study since Ethiopia has no national alcohol policy defining standard alcohol drinks [54]. Based on this, for a standard drink, 12 g of absolute alcohol was assumed which was considered as alcohol consumption. A standard drink was determined by converting local drinks to grams of pure alcohol, and then we specified the amount of pure alcohol per local drink and using local units of measure.

For this study, participants were categorized as abstainers or non-drinkers if women reported that they have never drunk any alcoholic beverages entirely throughout pregnancy, (AUDIT-C score = 0), low-risk drinkers, or non-hazardous drinkers if they reported 1 or 2 scores in AUDIT-C for the period of pregnancy) [55], and hazardous drinkers if they consumed a pattern or quantity of alcohol with an AUDIT-C score of three or more [56–58]. Different receptacles were used to measure local drinks, such as *‘tassa’, malekia’* and *‘birille’* for drinks *Tella* (traditional Ethiopian beer fermented from mostly barley but also with wheat, maize, sorghum, and mixed with ‘*Gesho’* [Rhamnusprinioides]) [33], *Areki* (a whiskey-like drink distilled from fermented barley or maize and mixed with ‘[Rhamnusprinioides]) and *Tej* (a honey wine), respectively. The amount of each drink consumed in ml was then calculated. This value was converted to grams of absolute alcohol by applying a conversion factor and taking into account the percentage of absolute alcohol present in each drink. Accordingly, a standard drink equivalent to 1 bottle beer (330 ml) at 5% x (strength) 0.79 (conversion factor) = 13 g of ethanol; 1 glass wine (140 ml) at 12% × 0.79 = 13.3 g of ethanol; 1 shot (*‘malekia’*) *Areki*(40 ml) at 40% × 0.79 = 12.6 g of ethanol, alcoholic content (30–50%); 1 *‘birille’ Tej* (200 ml) at 8% × 0.79 = 12.64 g of ethanol, alcoholic content *(*7–11%);and 1 *“tassa” Tella/Korofe* (330-500 ml) at 4.5% × 0.79 = 11.73 g of ethanol of alcoholic content (4–6%) [59–61].

### Data collection methods and tools

This data collection tool was similar to a previous article, a nested case-control study, which was part of this project published elsewhere [62]. The questionnaire was prepared first in English and then translated into Amharic (local language) to suit local applicability and then back to English to ensure its consistency. The tool was developed by reviewing different previous studies of similar objectives [2, 50, 51, 63–66], and then experts’ consultation was sought to ascertain its validity by considering the local situation of the study participants and clinical relevance. Data were collected using a standardized interviewer-administered questionnaire and reviewing maternal care logbooks at the health facilities. A detailed interview was done for each woman in private with a nurse or a midwife at the baseline using a pre-tested interviewer administrated questionnaire. Data collectors and supervisors trained on data collection tools, procedures during data collection, obtaining consent from participants, and not missing any questions in the questionnaire. The Amharic version questionnaire was pre-tested for clarity through a pilot study on 67 respondents in Bahir Dar town, which is 180 km away from the actual study area. The tool was checked for its reliability and validity before actual data collection. To assure data truthfulness, weekly meetings and daily supervision were conducted with supervisors and data collectors to observe the quality, status, and issues in collecting data. In addition to AUDIT-C, the tool also included the Edinburgh Postnatal Depression Scale (EPDS), which has 10 items scored on a scale of 0–3; the score ranging from 0 to 30, and we used a cut-off point of 13 and above on the scale to identify women with depressive symptoms [67].

The socio-economic status of the households (wealth index) was assessed using 16 variables extracted from Ethiopia Demographic and Health Survey 2016, and Principal Component Analysis was computed to determine it. The MUAC of the left arm with no clothing was measured in the third trimester using a flexible non-stretchable standard tape measure. Pregnant women having MUAC< 22 cm were considered undernourished and ≥ 22 cm normal [68]. When the hemoglobin level was less than 11.0 g/dl during the first or third trimester, the presence of anemia was considered [69].

### Statistical analysis

The data were entered using into EpiData 3.1.version and exported to STATA version 14. We computed bivariable log-binomial regression model analysis to see the association of selected maternal characteristics and prenatal alcohol use (non-hazardous and hazardous) with primary outcomes of interest using the chi-square test statistic. All variables significantly associated with outcomes of interest in bivariable analysis at *p*-value ≤0.2 were considered as candidates for the multivariable log-binomial regression model. In the final model, multivariable analysis was performed to examine the association between reported prenatal alcohol exposure (non-hazardous and hazardous) and interested adverse birth outcomes. The strength of associations of the regression model was reported using an adjusted risk ratio (ARR) with a corresponding 95% confidence interval (CI) and the *p*-value < 0.05 to declare the statistical significance threshold. The occurrence of multicollinearity among explanatory variables was ensured using the Variance Inflation Factor (VIF) at a cut-off point of 10 [70]. Complete case analyses were carried out by taking out any cases with a missing covariate. We used the adjusted PAR to determine the proportion of adverse birth outcomes that would not occur in a population if alcohol consumption were eliminated in the entire cohort after adjustment for potential confounders. This method first estimates the risk ratio for the alcohol consumption and for potential confounders and then estimates the number of events expected if the exposure of interest were eliminated to derive the percentage of outcomes attributable to alcohol consumption. The proportion of LBW, preterm birth, and stillbirth outcomes that could be attributed to maternal pregnancy alcohol consumption was estimated using Levin’s formula: PAR % = P*(ARR–1) / [P*(ARR–1) + 1] *100, where P was the proportion of women who used alcohol (hazardous or non-hazardous drinkers) during pregnancy, and ARR was the adjusted risk ratio of the adverse birth outcomes associated with the alcohol consumption [71, 72].

### Ethical considerations

Ethical clearance was obtained from the Institutional Ethical Review Board of the University of Gondar (R. No.-O/V/P/RCS/05/747/2019), and permission was received from Amhara Public Health Institute and Gondar town health department before the start of the study. Before enrolment of the participants, all respondents were informed about the importance of the study, its objective, effects, and the significance of participation. Verbal informed consent was also obtained before conducting data collection and all information was completed to maintain confidentiality. After taking the necessary information, all participants were counseled about the risks of alcohol drinking during pregnancy and advised not to drink any alcohol during pregnancy or while trying to get pregnant. Besides, women who engaged in hazardous drinking were referred to healthcare providers and proper linkage was established to get possible treatment options in their respective health facilities.

## Results

### Socio-demographic characteristics of the participants

A total of 1778 pregnant women were enrolled in the study, and of these, 1686 had data available on birth outcomes. We excluded 92 mother-infant pairs from the analysis due to missing appropriate birth outcome data (Fig. 1).

The mean maternal age at the baseline was 26.47 ± 4.58 years. Nearly two-thirds (60.8%) of the study participants were between the ages 25 and 34 years. Most of the women were married (97.4%) and were orthodox (88.2%). Three hundred sixty-six (21.7%) of them had no formal education, and only 510(30.2%) had tertiary education (Table 1).

**Table 1:** Socio-demographic and economic characteristics of study participants in Gondar town, Northwest Ethiopia, 2020 (*n* = 1686)

Variables	Intensity of alcohol consumption			Total (%)
	Non-drinkers *n* = 1122 (66.5%)	Non-hazardous *n* = 448 (79.4%)	Hazardous *n* = 116 (20.6%)	
**Health facilities**				
University of Gondar comprehensive specialized hospital	542(48.31)	239(53.35)	28(24.14)	809(47.98)
Gondar polyclinic	211(18.81)	132(29.91)	38(32.76)	383(22.79)
Azezo	229(20.41)	58(12.95)	42(36.21)	329(19.51)
Maraki	148(12.48)	17(3.79)	8(6.90)	165(9.79)
**Age group (years)**				
15–24	378(33.24)	141(31.03)	30(25.86)	549(32.56)
25–34	661(58.91)	281(62.72)	76(65.52)	1018(60.38)
≥ 35	83(7.40)	26(5.80)	10(8.62)	119(7.06)
**Marital status**				
Married	1094(97.50)	434(96.88)	114(98.28)	1642(97.39)
Single/divorced/separated/widowed	28(2.50)	14(3.13)	2(1.72)	44(2.61)
**Religion**				
Orthodox	933(83.16)	438(97.77)	116(100)	1487(88.20)
Muslim	179(15.95)	7(1.56)	0(0.00)	186(11.03)
Protestant	10(0.89)	3(0.67)	0(0.00)	13(0.77)
**Ethnicity**				
Amhara	1070(95.37)	433(96.65)	144(98.28)	1617(95.91)
Others	52(4.63)	15(3.35)	2(1.72)	69(4.09)
**Family size**				
1–2	417(37.17)	180(40.18)	46(39.66)	643(38.14)
3–4	574(51.16)	220(49.11)	61(52.59)	855(50.71)
≥ 5	131(11.68)	48(10.71)	9(7.76)	188(11.15)
**The educational level of respondents**				
No formal education	233(20.77)	101(22.54)	32(27.59)	366(21.71)
Primary education (Grade 1–8)	167(14.88)	63(14.06)	17(14.66)	247(14.65)
Secondary education(Grade 9–12)	385(34.31)	141(31.47)	37(31.90)	563(33.39)
Tertiary education(above Grade 12)	337(30.04)	143(31.92)	30(25.86)	510(30.25)
**Occupation**				
Housewife	525(46.79)	189(42.19)	64(55.17)	778(46.14)
Employed in any organization	233(20.77)	116(25.89)	32(27.59)	264(21.79)
Merchant	247(22.01)	122(27.23)	13(11.21)	382(22.66)
Students	75(6.68)	6(1.34)	1(0.86)	82(4.86)
Others	42(3.74)	15(3.35)	6(5.17)	63(3.74)
**Household wealth index**				
Poorest	260(23.17)	80(17.86)	9(7.76)	349(20.70)
Poor	225(20.05)	85(18.97)	33(28.45)	343(20.34)
Middle	198(17.65)	97(21.65)	20(17.24)	315(18.68)
Rich	225(20.05)	90(20.09)	26(22.41)	341(20.23)
Richest	214(19.07)	96(21.43)	28(24.14)	338(20.05)

### Reproductive and medical history related characteristics of the study participants

Almost half, (49.23%) of the study participants had one or two children. Concerning birth intervals, the majority (81.91%) of pregnant women had 24 months and above birth intervals. More than half, (60.50%) of the women had experienced at least one previous birth (multiparous). Two hundred ten (12.46%) of the respondents had experienced a history of abortion. Among the study participants who were tested for hemoglobin level, 248(14.86%) of the pregnant women had anemia (Table 2).

**Table 2:** Reproductive and medical history related characteristics of study participants attending ANC at public health facilities in Gondar town, Northwest Ethiopia, (*n* = 1686)

Variables	The intensity of alcohol consumption			Total (%)
	Non-drinkers *n* = 1122 (66.5%)	Non-hazardous *n* = 448 (79.4%)	Hazardous *n* = 116 (20.6%)	
**Sex of the newborn**				
Male	543(48.40)	218(48.66)	51(43.97)	812(48.16)
Female	579(51.60)	230(51.34)	65(56.03)	874(51.84)
**Number of children**				
No child yet	446(39.75)	189(42.19)	45(38.79)	680(40.33)
1–2 children	544(48.48)	223(49.78)	63(54.31)	830(49.23)
≥ 3	132(11.76)	36(8.04)	8(6.90)	176(10.44)
**Birth interval (*n*** **= 1006)**				
< 24 months	145(21.14)	28(11.20)	9(12.86)	182(18.09)
≥ 24 months	541(78.86)	222(88.80)	61(87.14)	824(81.91)
**MUAC**				
< 22 cm	143(12.75)	84(18.75)	26(22.41)	253(15.01)
≥ 22 cm	979(87.25)	364(81.25)	90(77.59)	1433(84.99)
**History of abortion**				
Yes	127(11.32)	66(14.73)	17(14.66)	210(12.46)
No	995(88.68)	382(85.27)	99(85.34)	1476(87.54)
**History of preterm birth (*n*** **= 1020)**				
Yes	28(4.14)	13(4.71)	7(9.72)	48(4.71)
No	648(95.86)	259(95.22)	65(90.28)	972(95.29)
**Depression**				
Yes	195(17.38)	71(15.85)	44(37.93)	310(18.39)
No	927(82.62)	377(84.15)	72(62.07)	1376(81.61)
**History of known diabetes mellitus**				
Yes	12(1.07)	5(1.12)	3(2.59)	20(1.19)
No	1110(98.93)	443(98.88)	113(97.41)	1666(98.81)
**Anemia(*n*** **= 1669)**				
Yes	147(13.21)	62(13.96)	39(34.82)	248(14.86)
No	966(86.79)	382(86.04)	73(65.18)	1421(85.14)
**Hypertensive disorders in pregnancy**				
Yes	88(7.84)	55(12.28)	20(17.24)	163(9.67)
No	1034(92.16)	393(87.72)	96(82.76)	1523(90.33)
**Drinking coffee**				
Yes	839(74.78)	331(73.88)	86(74.14)	1256(74.50)
No	283(25.22)	117(26.12)	30(25.86)	430(25.50)
**Smoking**				
Yes	2(0.18)	2(0.45)	2(1.72)	6(0.36)
No	1120(99.82)	446(99.55)	114(98.28)	1680(99.64)
**Khat chewing**				
Yes	15(1.34)	8(1.79)	2(1.72)	25(1.48)
No	1107(98.66)	440(98.21)	114(98.28)	1661(98.52)

### Alcohol consumption during pregnancy

Among women who used alcohol of the study participants, approximately one-fifth (20.57%) reported taking hazardous alcohol during their current pregnancy. Five hundred thirty-eight (95.39%) of the alcohol drinkers used alcoholic beverages in the first trimester, 548(97.16%) and 539(95.57%) consumed in the second and third trimesters, respectively.

Regarding the amount of alcohol consumption, 98(17.38%) of the respondents used six or more drinks on one occasion during their current pregnancy. Likewise, most pregnant women (68.62%) consumed one or two standard drinks, 21.10% had three or four drinks, and 10.28% of the participants had five or more standard drinks on a typical day. Concerning the types of alcoholic beverages that were consumed by respondents, the most commonly used alcoholic beverage in the first trimester was *Tella*(56.03%), followed by beer/draft (19.15%). In the second trimester, 39.18, 31.74, 3.55, 2.30, and 20.39% of the respondents consumed beer/draft, *Tella,* wine, A*reki/Tej/Korofe, and* two or more types of drinks, respectively. Finally, types of alcoholic beverages which were consumed in the third trimester were beer/draft (35.64%), *Tella* (31.21%), *Areki/Korofe/Tej/*wine (5.33%), and two or more types of drinks (23.94%) (Table 3).

**Table 3:** Alcohol intake during pregnancy using Alcohol Use Disorders Identification Test- Consumption (AUDIT-C)

Variables	Number (percent)
**How often do you have a drink containing alcohol during your current pregnancy (*n*** **= 1686)**	
(0) Never	1122(66.55%)
(1) Monthly or less	345(61.17%)
(2) 2 to 4 times a month	151(26.77%)
(3) 2 to 3 times a week	66(11.70%)
(4) 4 or more times a week	2(0.35%)
**How many drinks containing alcohol do you have on a typical day when you are drinking during pregnancy? (*n*** **= 564)**	
(0) 1 or 2	387(68.62%)
(1) 3 or 4	119(21.10%)
(2) 5 or 6	43(7.62%)
(3) 7, 8, or 9	15(2.66%)
(4) 10 or more	0(0.00%)
**How often do you have six or more drinks on one occasion during your current pregnancy? (*n*** **= 564)**	
(0) Never	466(82.62%)
(1) Less than monthly	67(11.88%)
(2) Monthly	29(5.14%)
(3) Weekly	2(0.35%)
(4) Daily or almost daily	0(0.00%)

### Birth outcomes

The mean birth weight of newborns who were delivered from singleton pregnancies was 2994.68 ± 433.02 g, and the gestational age of newborns was 38.84 ± 1.66 weeks. The incidence of low birth weight in the whole cohort (weighed less than the 2500 g) was 12.63% (95% CI: 11.12, 14.31). Likewise, the incidence of preterm (< 37 weeks gestation) and stillbirth were 6.05% (95% CI: 5.00, 7.29) and 4.27% (95% CI: 3.4, 5.35), respectively.

### Relationship between the level of alcohol intake and birth outcomes

Overall, there was some difference between the adverse birth outcomes for infants of mothers who drank any levels of alcohol during pregnancy and women who were abstinent during pregnancy. Alcohol consumption during pregnancy had dose-response relationship with the risk of low birth weight (10.25% for no consumption, 15.18% for non-hazardous, and 25.86% for hazardous consumption at chi-square = 26.80, *P* < 0.001) and preterm delivery (5.53% for no consumption, 5.58% for non-hazardous, 12.93% for hazardous consumption at chi-square = 10.38, *P* < 0.001). On the other hand, there was an inconsistency of dose-response relationship for stillbirth between babies born to mothers of alcohol-consuming and women who did not (4.28% for no consumption, 3.13% for non-hazardous, 8.62% for hazardous consumption at chi-square = 6.81, *P* = 0.033).

Women who reported a hazardous pattern of alcohol intake during pregnancy were 2.34 times (ARR = 2.34; 95% CI: 1.66, 3.30) increased the risk of low birth weight when compared to women who abstained entirely throughout pregnancy. Similarly, the risk of LBW was 50% (ARR = 1.50; 95% CI: 1.31, 1.98) higher for non-hazardous alcohol drinker pregnant women when compared to women who did not consume any alcohol. Analysis of the hazardous level of alcohol consumption during pregnancy yielded 2.06 times (ARR = 2.06; 95% CI: 1.21, 3.52) increased the risk of preterm birth compared to abstinent during pregnancy, but the association was not observed at non-hazardous levels of alcohol use during pregnancy (Table 4). The adjusted PAR of low birth weight related to non-hazardous and hazardous alcohol drinking during pregnancy was 11.72 and 8.44%, respectively, while the adjusted PAR of hazardous alcohol consumption was 6.80% for preterm.

**Table 4:** Associations between alcohol consumption during pregnancy, and some maternal characteristics and adverse fetal outcomes at public health facilities in Gondar town, Northwest, Ethiopia, 2020

Variables	Low birth weight		RR (95% CI)	ARR (95% CI)	Preterm		RR (95% CI)	ARR (95% CI)
	Yes	No			Yes	No		
**Age of the mother**								
15–24	91(16.58)	458(83.42)	1	1	44(8.01)	505(91.99)	1	1
25–34	108(10.61)	910(89.39)	0.64(0.49, 0.83)	0.80(0.59,1.08)	48(4.72)	970(95.28)	0.59(0.40, 0.87)	0.68(0.43,1.07)
≥ 35	14(11.76)	105(89.24)	0.71(0.42, 1.20)	0.89(0.47,1.67)	10(8.40)	109(91.60)	1.05(0.54, 2.02)	0.98(0.40,2.20)
**Education level**								
No formal education	40(10.93)	326(89.07)	0.94(0.64, 1.57)	**1.50(1.01,2.23)**	35(9.56)	331(90.44)	2.32(1.38,3.92)	1.45(0.78,2.68)
Primary education	40(16.19)	207(83.81)	1.40(0.97, 2.03)	1.34(0.92,1.94)	18(7.29)	229(92.71)	1.77(0.96, 3.26)	0.98(0.49,1.99)
Secondary education	74(13.14)	489(86.86)	1.14(0.82, 1.57)	1.44(0.92,2.25)	28(4.97)	535(95.03)	1.21(0.69, 2.10)	0.80(0.43,1.48)
Tertiary education	59(11.57)	451(88.43)	1	1	21(4.12)	489(95.88)	1	1
**Occupation**								
Merchant	19(13.97)	117(86.03)	1	1	10(2.62)	372(97.38)	1	1
Housewife	56(20.97)	211(79.03)	1.44(1.02,2.02)	1.83(0.83,1.68)	69(8.78)	709(91.13)	3.39(1.77,6.50)	**2.87(1.47,5.62)**
Employed in any organization	20(13.07)	133(86.93)	1.05(0.70,1.60)	0.92(0.59,1.43)	14(3.67)	367(96.33)	1.40(0.63,3.12)	1.40(0.61,3.24)
Others	5(17.86)	23(82.14)	1.28(0.77,2.15)	1.09(0.64,1.84)	9(6.21)	136(93.79)	2.37(0.98,5.72)	1.98(0.97,2.34)
**Household wealth status**								
Poorest	39(11.17)	310(88.83)	1	1	–	–	–	**–**
Poor	54(15.74)	289(84.26)	1.4(0.96, 2.07)	1.26(0.86,1.85)	–	–	–	**–**
Middle	20(6.35)	295(93.65)	0.57(0.34, 0.95)	**0.57(0.34,0.96)**	–	–	–	**–**
Rich	49(14.37)	292(85.63)	1.29(0.87, 1.91)	1.23(0.84,1.83)	–	–	–	**–**
Richest	51(15.09)	287(84.91)	1.35(0.91, 1.99)	1.05(0.69,1.59)	–	–	–	**–**
**Family size**								
1–2	97(15.09)	546(84.91)	1	1	50(7.78)	593(92.22)	1	1
3–4	94(10.99)	761(88.01)	0.73(0.56, 0.95)	**1.86(1.24,2.80)**	38(4.58)	817(95.56)	0.57(0.38, 0.86)	0.73(0.34,1.57)
5 and above	22(11.70)	166(88.30)	0.76(0.50, 1.19)	**2.21(1.21,4.00)**	14(7.45)	174(92.55)	0.96(0.54, 1.69)	1.00(0.33,3.01)
**Parity**								
Nulliparous	113(16.97)	553(83.03)	1.73(1.35,2.22)	**2.53(1.68,3.82)**	–	–	–	–
Multiparous	100(9.8)	920(90.20)	1	1	–	–	–	–
**Number of children**								
No child yet	–	–	–	–	51(7.50)	629(92.50)	1	1
1–2	–	–	–	–	38(4.58)	792(95.42)	0.61(0.41, 0.92)	0.96(0.43,2.12)
≥ 3	–	–	–	–	13(7.39)	163(92.61)	0.98(0.55, 1.76)	1.06(0.33,3.41)
**Status of pregnancy**								
Planned	176(12.07)	1282(87.93)	1	1	–	–	–	–
Un planned	37(16.23)	191(83.77)	1.34(0.97,1.86)	1.23(0.89,1.71)	–	–	–	–
**Hypertensive disorders of pregnancy**								
Yes	–	–	–	**–**	14(8.59)	149(91.41)	1.50(0.87, 2.55)	**1.97(1.14,3.39)**
No	–	–	–	**–**	88(5.78)	1435(94.22)	1	1
**Alcohol consumption**								
Hazardous	30(25.86)	86(74.14)	2.52(1.77, 3.59)	**2.34(1.66, 3.30)**	15(12.93)	101(87.07)	2.34(1.38, 3.98)	**2.06(1.21,3.52)**
Non- hazardous	68(15.18)	380(84.82)	1.48(1.12, 1.96)	**1.50(1.31,1.98)**	25(5.58)	423(94.42)	1.01(0.64, 1.59)	1.03(0.66,1.62)
Non-drinker	115(10.25)	1007(89.75)	1	1	62(5.53)	1060(94.47)	1	1
**Sex of newborn**								
Male	–	–	–	–	62(7.64)	750(92.36)	1	1
Female	–	–	–	–	40(4.58)	834(95.42)	1.67(1.13,2.45)	**1.55(1.05,2.27)**
**Anemia**								
Yes	48(19.35)	200(80.65)	1.69(1.26, 2.26)	**1.65(1.24,2.21)**	–	–	–	–
No	163(11.47)	1258(88.53)	1	1	–	–	–	–
**Advise the risks of alcohol use during ANC visit**								
Yes	37(9.05)	372(90.95)	1	1	–	–	–	–
No	176(13.78)	1101(86.22)	1.52(1.09, 2.13)	1.32(0.94,1.84)	–	–	–	–
**MUAC**								
< 22 cm	48(18.75)	208(81.25)	1.63(1.21, 2.18)	**1.47(1.09,1.97)**	24(8.98)	232(90.63)	1.72(1.11, 2.66)	1.51(0.97,2.34)
≥ 22 cm	165(11.54)	1265(88.46)	1	1	78(5.45)	1352(94.55)	1	1

Before adjusting for potential confounders, the association between stillbirth and the hazardous level of alcohol consumption was found to be two-fold (RR = 2.01; 95% CI: 1.05, 3.88) higher than abstinent during pregnancy. However, there was no evidence of an increased likelihood of stillbirth at any levels of alcohol consumption during pregnancy after adjustment for other covariates (Table 5).

**Table 5:** Associations between alcohol consumption during pregnancy, and some maternal characteristics and stillbirth at public health facilities in Gondar town, Northwest, Ethiopia, 2020

Variables	Stillbirth		RR (95%CI)	ARR (95% CI)
	Yes	No		
**Family size**				
1–2	37(5.75)	606(94.25)	1	1
3–4	31(3.63)	824(96.37)	0.63(0.39, 1.00)	0.44(0.18,1.07)
5 and above	4(2.13)	184(97.87)	0.37(0.13, 1.02)	**0.23(0.06,0.83)**
**Parity**				
Nulliparous	35(5.26)	631(94.74)	1.45(0.92,2.27)	0.61(0.25,1.47)
Multiparous	37(3.63)	983(96.37)	1	1
**Alcohol consumption**				
Hazardous	10(8.62)	106(91.38)	2.01(1.05, 3.88)	1.64(0.84,3.22)
Non- hazardous	14(3.13)	434(96.88)	0.73(0.41,1.31)	0.72(0.40,1.29)
Non-drinker	48(4.28)	1074(95.72)	1	1
**Anemia**				–
Yes	17(6.85)	231(93.15)	1.77(1.05, 3.00)	**1.72(1.00,2.96)**
No	55(3.87)	1366(96.13)	1	1
**Preterm birth**				
Yes	12(11.76)	90(88.24)	3.11(1.73, 5.58)	**2.99(1.65,5.41)**
No	60(3.79)	1524(96.21)	1	1
**Birth weight**				
< 2500 g	13(6.10)	200(93.90)	1	1
≥ 2500 g	59(4.01)	1414(65.99)	152(0.85,2.73)	1.14(0.63,2.10)

## Discussion

To the best of our knowledge, this is the first prospective cohort study regarding maternal alcohol consumption in Ethiopia that looked at hazardous and non-hazardous alcohol exposures during pregnancy separately and their association with adverse birth outcomes. Maternal alcohol consumption during pregnancy in many countries continues to be the single most important modifiable risk factor for adverse birth outcomes. This study examined the potential effects of alcohol (hazardous and non-hazardous) consumption during pregnancy on adverse birth outcomes. In the present study, we found the risk of low birth weight significantly increased among newborns of mothers who drank both hazardous and non-hazardous alcohol during pregnancy. Likewise, there was a statistically significant association between hazardous alcohol intake and preterm birth. However, there was no evidence of the association between alcohol consumption during pregnancy and stillbirth had been observed after taking into account other covariates. In addition to alcohol consumption, other risk factors associated with low birth weight: education level, household wealth status, family size, anemia in pregnancy, and MUAC; and preterm birth: occupation, hypertensive disorder of pregnancy, and sex of the newborn. Stillbirth was associated with family size, anemia, and preterm birth.

Findings from our study showed that an increasing trend in the risk of low birth weight with increasing levels of alcohol exposure was statistically significant. In this cohort study, prenatal hazardous and non-hazardous alcohol exposures were 2.34 times and 50% more likely to increase the risk of low birth weight compared to abstainers, respectively. On the other hand, the analyses of adjusted PAR percent also showed that 8.44and 11.72% of low birth weight cases might be attributed to hazardous and non-hazardous alcohol consumption, respectively. Some studies have concordantly found that maternal prenatal alcohol exposure was negatively associated with the weight of newborns [37, 73, 74]. Similarly, our study is consistent with several studies examining higher levels of prenatal alcohol consumption that have been linked with low birth weight [1, 9, 39]. Simultaneously, non-hazardous or light to moderate alcohol consumption during pregnancy had a positive significant association with the risk of low birth weight. This finding agreed with previous results of other studies that found low weight in newborns that were prenatally exposed to low to moderate maternal drinking [75–77]. The reason for this association could be justified that alcohol consumption during pregnancy impairs placental growth, lead to vasoconstriction, and interferes placental transmission of necessary nutrients and sufficient oxygen to the fetus [78]. On the contrary, some studies conducted in various areas detected no association between non-hazardous alcohol consumption and low birth weight [1, 26]. The possible explanation for this disagreement might be the difference in genetic material or biological variation for alcohol absorption and metabolism in the mothers and their fetus, data collection tools or methods, a cut-off value for low to a moderate level, characteristics of the study participants, lack of consistent definition for non-hazardous or low to moderate alcohol use and timing of consumption [79, 80]. Another potential reason for variation could be the heterogeneity of the quality of alcohol consumed; in our study, the majority of alcoholic drinks were locally prepared or homemade, which are prone to contamination with methanol and has high alcohol-related harm [12, 81]. Also, there might be the failure to adjust appropriately or differences in adjustment for possible confounding covariates between the studies, and residual confounding might have been a problem in some studies because of incorrectness in measuring confounders.

Similarly, based on our analysis, alcohol consumption during pregnancy has a dose-response relationship with preterm birth. We have attempted to provide the public health burden of hazardous alcohol consumption for preterm by quantifying PAR percent. We also found an association of hazardous prenatal alcohol drinking with preterm delivery compared to abstainers during pregnancy after adjusting for possible confounding factors. The finding showed that preterm birth was higher among women who reported hazardous alcohol consumption (AUDIT-C score ≥ 3) implies that exposure to excess alcohol during pregnancy has the potential to premature delivery. This result is consistent with the findings of other earlier studies that found preterm birth in neonates who were prenatally exposed to hazardous or heavy maternal drinking [38, 82]. The possible clarification for this link could be due to the association of prenatal alcohol exposure with placental dysfunction, diminished placental size, impaired blood flow and important nutrient transportation, and endocrine changes, any of which could play a role in the alcohol exposure effects on preterm birth [78]. On the other hand, the present finding is not in line with the result of other studies [76]. Our study could not find the effect of non-hazardous alcohol consumption on the risk for preterm delivery. This finding is consistent with previous studies of low to moderate alcohol exposure [83–85] and a systematic review of low-to-moderate alcohol consumption [26]. However, our result lacked the concordance with a prospective cohort study among mother-child pairs that demonstrated a light and mild level of maternal alcohol intake during pregnancy was positively associated with the risk of preterm birth [86]. The possible explanations for the discrepancy of our finding with earlier studies could be methods of assessment of maternal alcohol intake during pregnancy and variation in classifications of alcohol consumption (e.g. we categorized as non-drinker or abstainer, non-hazardous and hazardous based on AUDIT-C score, but others might not be similar to this). On the other hand, our study participants were not identical to the study participants of the previous studies with respect to biology and other characteristics that might cause the difference in susceptibility to adverse effects of alcohol use.

On the other analysis, there was no statistically significant association between any levels of alcohol consumption during pregnancy and stillbirth. This finding is consistent with the studies conducted in various parts of the world [87]. However, there are some controversial findings in the relationship between alcohol consumption during pregnancy and stillbirth; they confirmed that there was a positive link between higher threshold prenatal alcohol exposure and stillbirth [88–90]. This lack of relationship might be due to the limited information received about dose and frequency of alcohol consumption, unobserved heterogeneity among the study participants, and differences in exposure ascertainment that make it difficult to compare our results with those of findings. Generally, the variations between our findings and other studies could be to some extent due to heterogeneity between studies related to the method of alcohol assessments and inconsistent choice of potential confounders. Furthermore, the discrepancy in findings between nations may be a reflection of differences in alcoholic beverages and drinking patterns. Lastly, differences might also be due to genetic variations linked to the metabolism of alcohol that may differ between populations [91].

### Strengths and limitations of the study

One of the strengths of this study is the clarification of a dose-response relationship between maternal prenatal alcohol consumption and adverse birth outcomes, including locally brewed alcohols. It is important that only limited evidence exists on the effect of non-hazardous alcohol levels of prenatal alcohol exposure on adverse birth outcomes. Another strength of this study is determining population attributable risk, which allows public health programmers to address what percent of adverse birth outcomes could be prevented if alcohol consumption during pregnancy were to be taken out from the pregnant women.

Despite these strengths, due to the presence of some limitations, the findings of this study should be interpreted with caution. Alcohol consumption information was collected based on maternal self-report and hence is subject to recall bias. Women who consumed alcohol were more likely to either falsely refuse the alcohol use or significantly underreport the actual level that they drank and then could be categorized as non-drinker because drinking alcohol during pregnancy is considered socially unacceptable [92]. Thus, the reported amounts of alcoholic beverages consumed may be considerably lower than the real value biasing the study results due to misclassification and would under-estimate the true link between drinking and adverse birth outcomes, leading to a type II error. Nevertheless, self-report has been found to be more precise than other methods [93]. Most of the alcohol used was locally homemade brews; therefore, the exact volume of containers of alcohol was not well understood by the respondents, so it was difficult to get the factual standard drink during conversion.

## Conclusions and recommendations

Our findings suggest that there is an increasing risk of adverse birth outcomes, particularly preterm delivery and low birth weight, with increasing the level of alcohol intake. This result showed that the prevention of maternal alcohol use during pregnancy has the potential to reduce low birth weight and preterm. Hence, screening women for alcohol use during ANC visits and provide advice with rigorous follow-up of women who used alcohol may save the fetus from the potential risks of adverse birth outcomes. Healthcare workers have maintained strong and consistent messages of alcohol abstinence for pregnant women. Healthcare professionals should always be supported by comprehensive and up-to-date information on prenatal alcohol use and incorporate such information to prevent alcohol use among women before they become pregnant [94]. The lack of an association between prenatal alcohol exposure and stillbirth in this study needs further investigation.

## Data Availability

The datasets used and/or analyzed during the current study are available from the corresponding author on reasonable request.

## Abbreviations

ANC: Antenatal Care
AUDIT: Alcohol Use Disorders Identification Test
AUDIT-C: Alcohol Use Disorder Identification Test-Consumption
EPDS: Edinburgh Postnatal Depression Scale
FASD: Fetal Alcohol Spectrum Disorder
LAMP: Last Menstrual Period
MUAC: Mid-Upper Arm Circumference
PAR: Population-Attributable Risk
VIF: Variance Inflation Factor
WHO: World Health Organization
